# Supplementary material for: Three-dimensional liquid metal-based neuro-interfaces for human hippocampal organoids
Source: Nat Commun. 2024 May 14;15:4047. doi: 10.1038/s41467-024-48452-5 (PMC11094048; doi:10.1038/s41467-024-48452-5)
Supplement: Supplementary file 1 — Supplementary Information [file 41467_2024_48452_MOESM1_ESM.pdf]

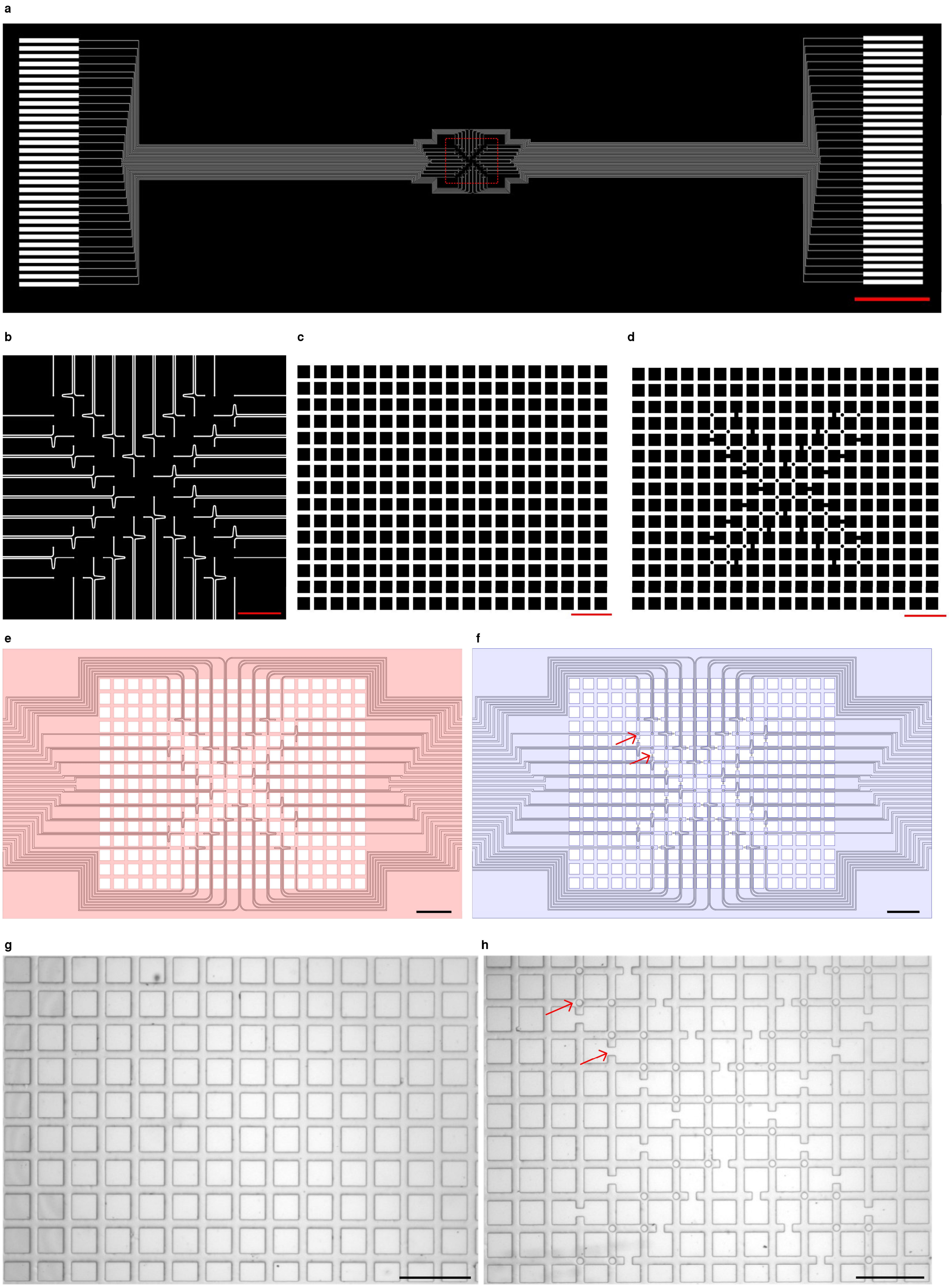

Supplementary Fig. 1 | Design of PDMS patterns. a-d, Photomask designs used in photolithography for the conductor (**a** and **b**, Figure **b** is the enlarged view of the red dashed box in Figure **a**), the bottom substrate (**c**), and the top insulating (**d**) layers. The white area represented the exposure area of photolithography and was also the location of microchannels on PDMS slabs. **e-f**, AutoCAD plotting of the alignment between different layers. (**e**): the conductor and the bottom substrate layers; (**f**): the conductor and the top insulating layers. **g-h,** PDMS slabs for patterning the bottom substrate (**g**) and the top insulating (**h**) layers. Breakpoints and circles in the mesh (red arrows in Figures **f** and **h**) represented pillars that prevent the PU solution from covering MPC electrodes. Scale bar: 5 mm (**a**) and 500 μm

(others).

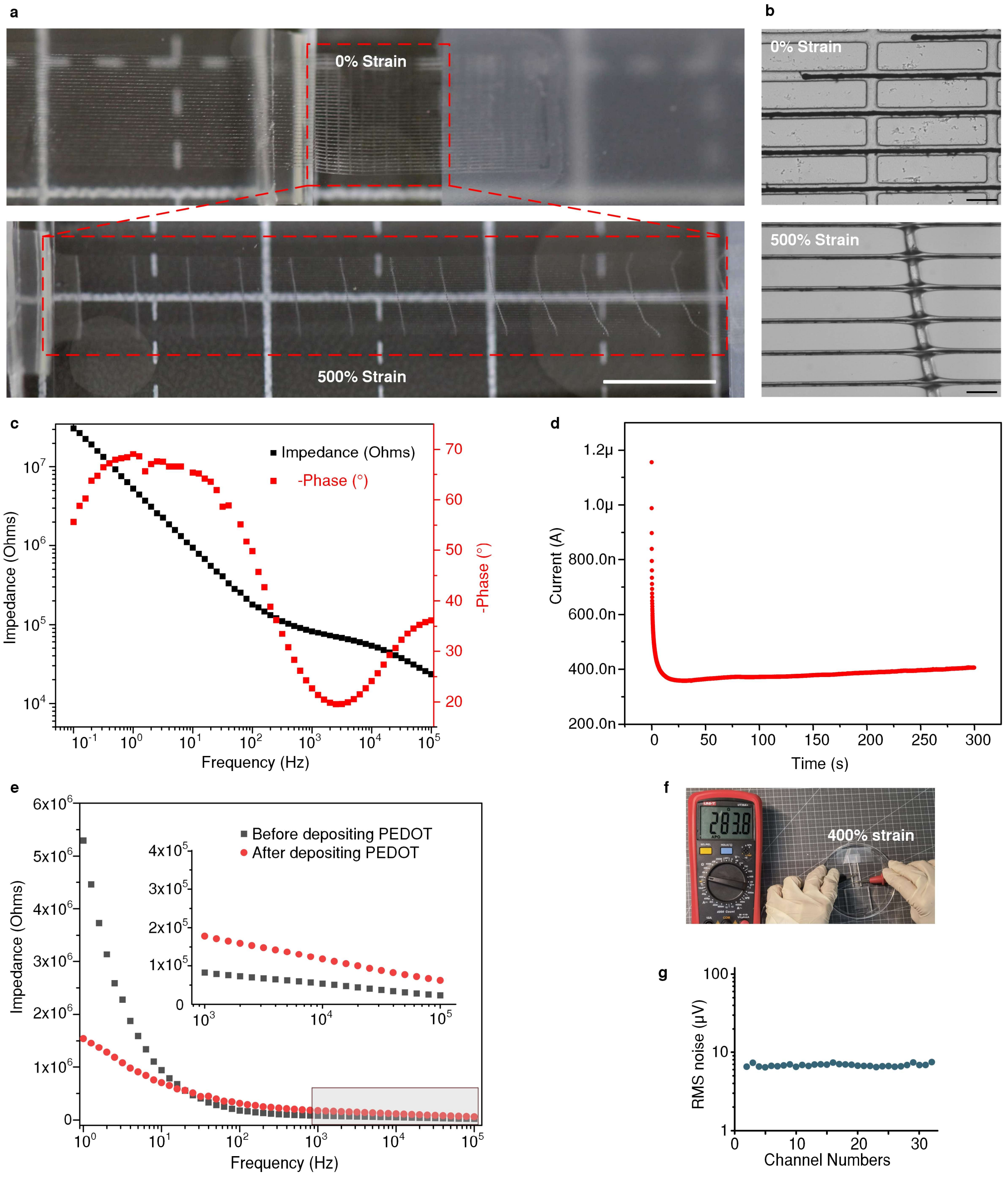

Supplementary Fig. 2 | Elongation of mesh MPC and electrodeposition of PEDOT. a, Images of a mesh MPC with 500% elongation. Scale bar: 5 mm. **b,** MPC circuits with 30 μm width before and after 500% strain. Scale bar:100 μm. **c,** Impedance and phase dependence on the frequency before coating PEDOT. **d,** Current versus time curve during the deposition of PEDOT under the constant potential at 1.2 V. **e,** Impedance across frequency before and after coating PEDOT. The inset shows magnified plots of the grey translucent box from 1 kHz to 100 kHz. **f,** The conductivity under 400% strain. **g,** RMS noise of 32 electrodes in the culturing medium. RMS, root mean square.

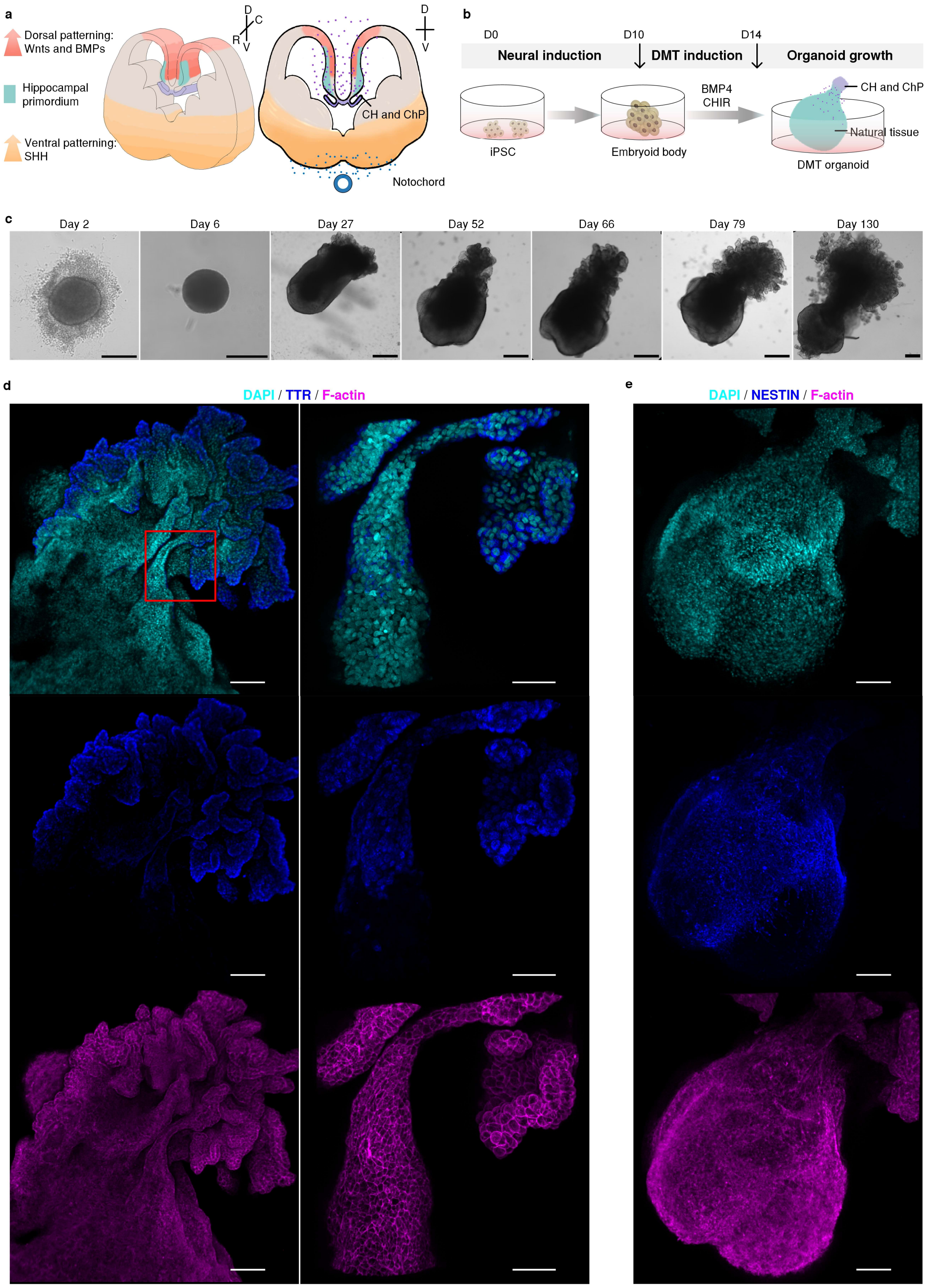

Supplementary Fig. 3 | DMT organoids. a, Schematics diagram showing the generation of the *in vivo* hippocampal primordium from DMT. The CH and ChP release Wnts and BMPs, and the notochord releases SHH. **b,** Induction process of DMT organoids that form CH, ChP, and neural tissue. **c,** The typical morphology of DMT organoids at different ages. Two parts appeared in DMT organoids. One part looked like 'leaves.' They were ChP epithelial cells (see figure **d**). Another part looked like a 'trunk.' It was neural tissue (see figure **e**). Scale bar: 100 μm in figures labeled 'Day 2' and 'Day 6', 200 μm in other figures. **d,** Left column: TTR$^+$ ChP epithelial cells in the DMT organoids. Scale bar: 150 μm. TTR is the marker of

ChP epithelial cells, mainly expressed in the loose 'leaves' part of DMT organoids. Right column: zoom-in view of the red box in the left figure showing the apparent boundary of TTR⁺ epithelial cells. Scale bar: 50 μm. **e,** Images of NESTIN⁺ neural tissue in the DMT organoid, mainly expressed in the spherical 'trunk' part. Scale bar: 150 μm. DMT, dorsomedial telencephalon.

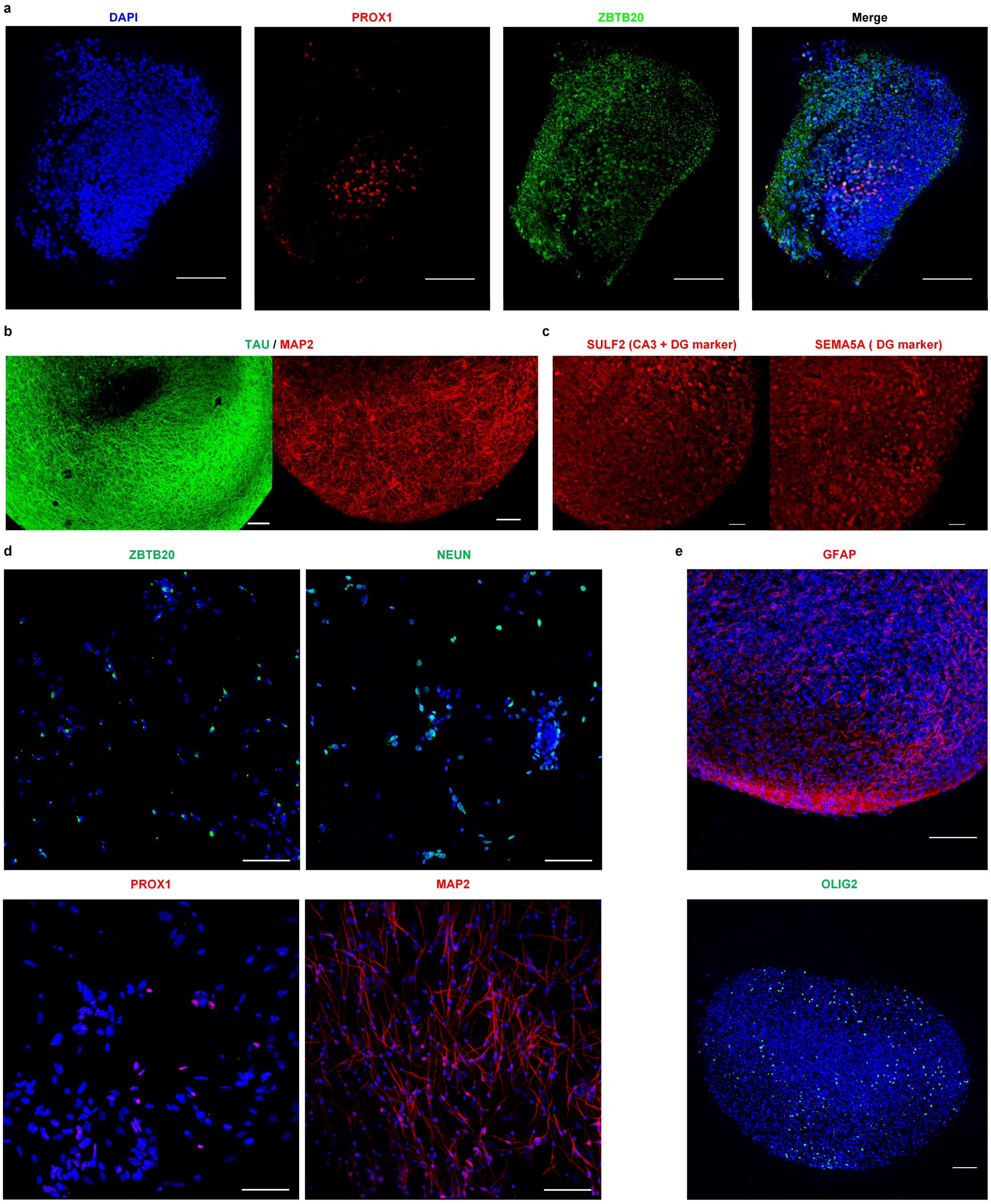

Supplementary Fig. 4 | HHOs. a, ZBTB20⁺PROX1⁺ granule neurons in the day-85 hHO. Scale bar: 100 μm. **b,** Neuronal marker TAU and MAP2 were expressed in hHOs. Scale bar: 100 μm. **c,** The hippocampal markers SULF2 and SEMA5A, were expressed in hHOs. Scale bar: 40 μm. **d,** The expression of hippocampal markers ZBTB20 and PROX1, and neuron markers NEUN and MAP2 in the cells dissociated from hHOs. Scale bar: 100 μm. **e,** The expression of astrocyte marker GFAP and oligodendrocyte progenitor cell marker OLIG2 in hHOs. Scale bar: 100 μm.

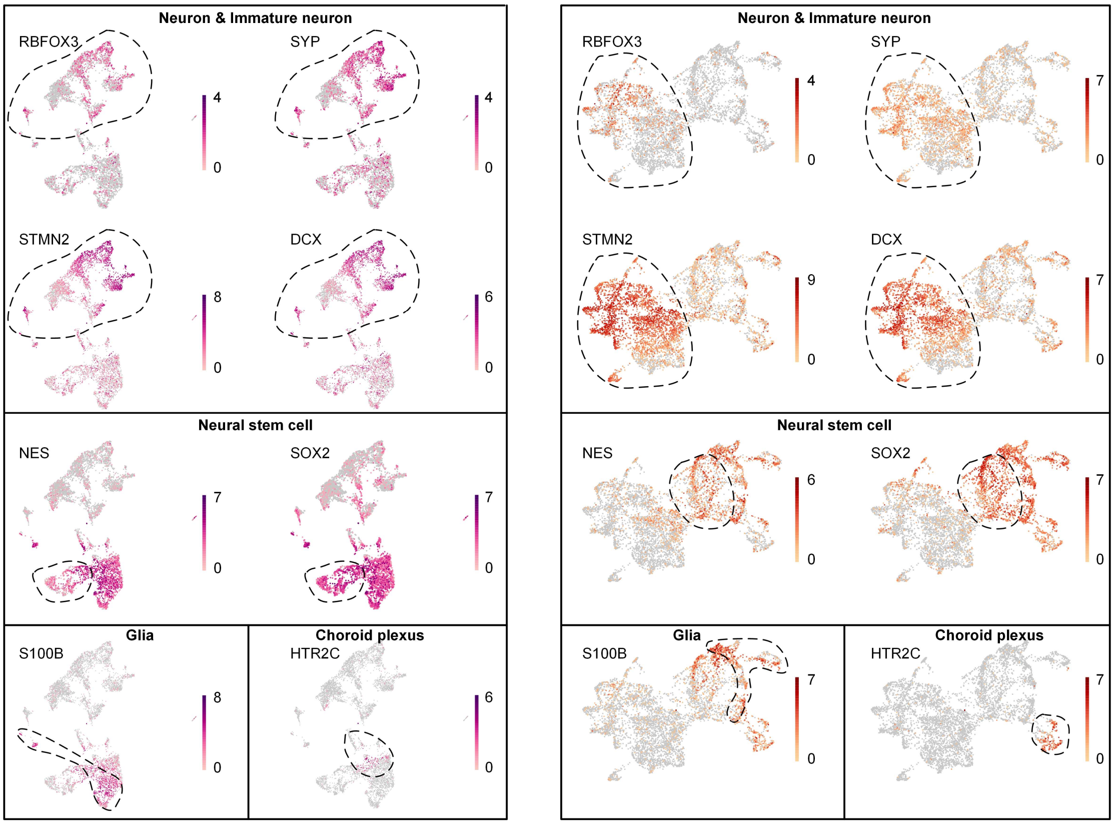

Supplementary Fig. 5 | Similar cell types in two samples of hHOs. (Left) UMAP visualization of day-70 hHOs. (Right) UMAP visualization of day-81 hHOs.

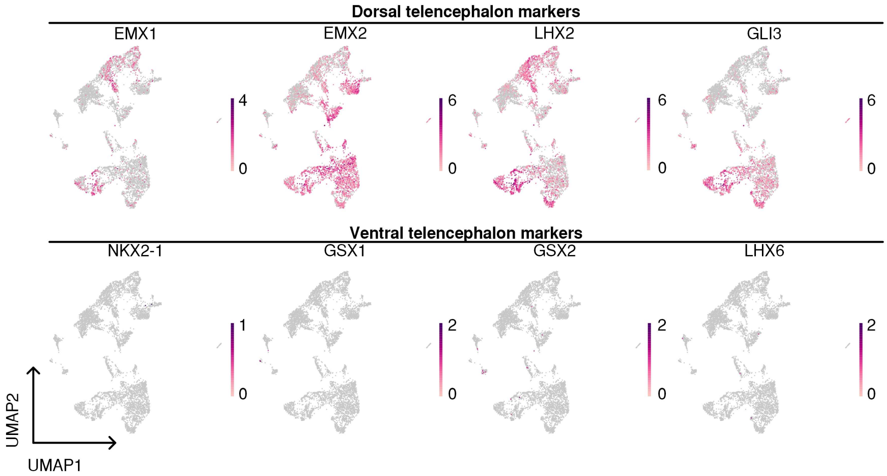

Supplementary Fig. 6 | Expression of dorsal and ventral telencephalon markers in day-70 hHOs.

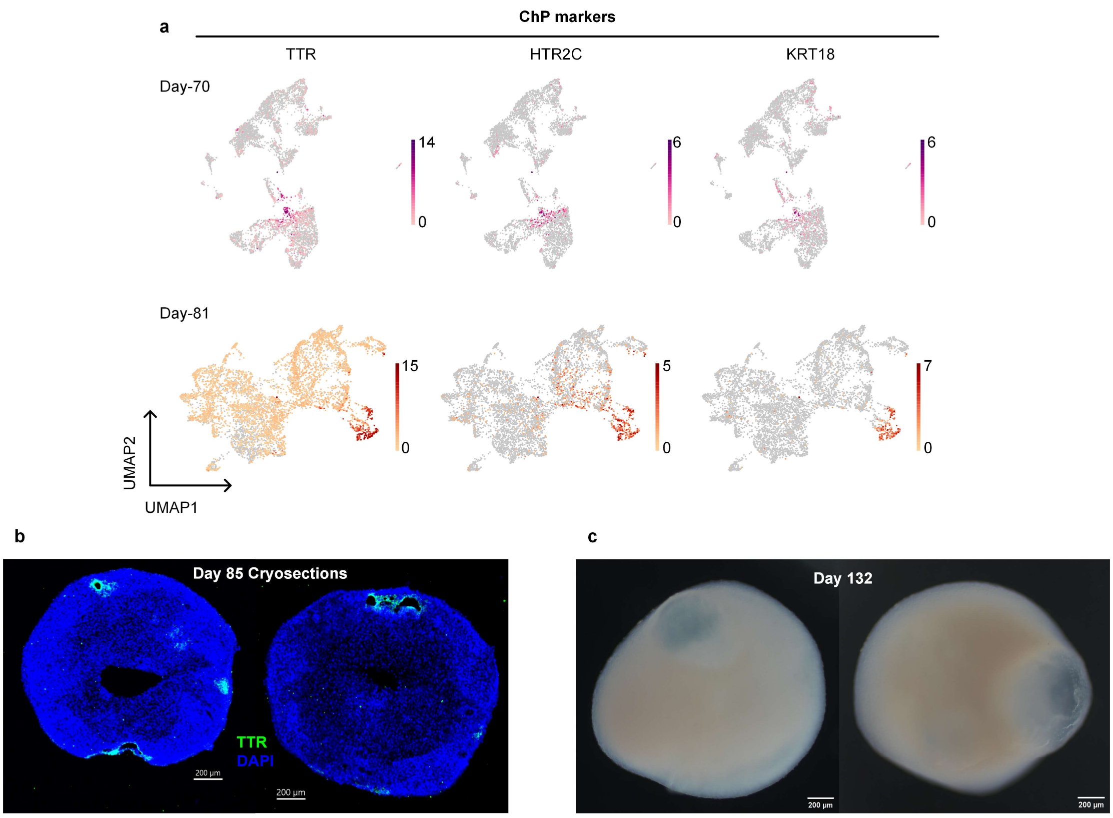

Supplementary Fig. 7 | ChP in hHOs. a, UMAP visualization of the ChP cluster in day-70 and day-81 hHOs. **b,** The expression of TTR in two cryosections of the day-85 hHO. **c,** Cavities appeared in day-132 hHOs.

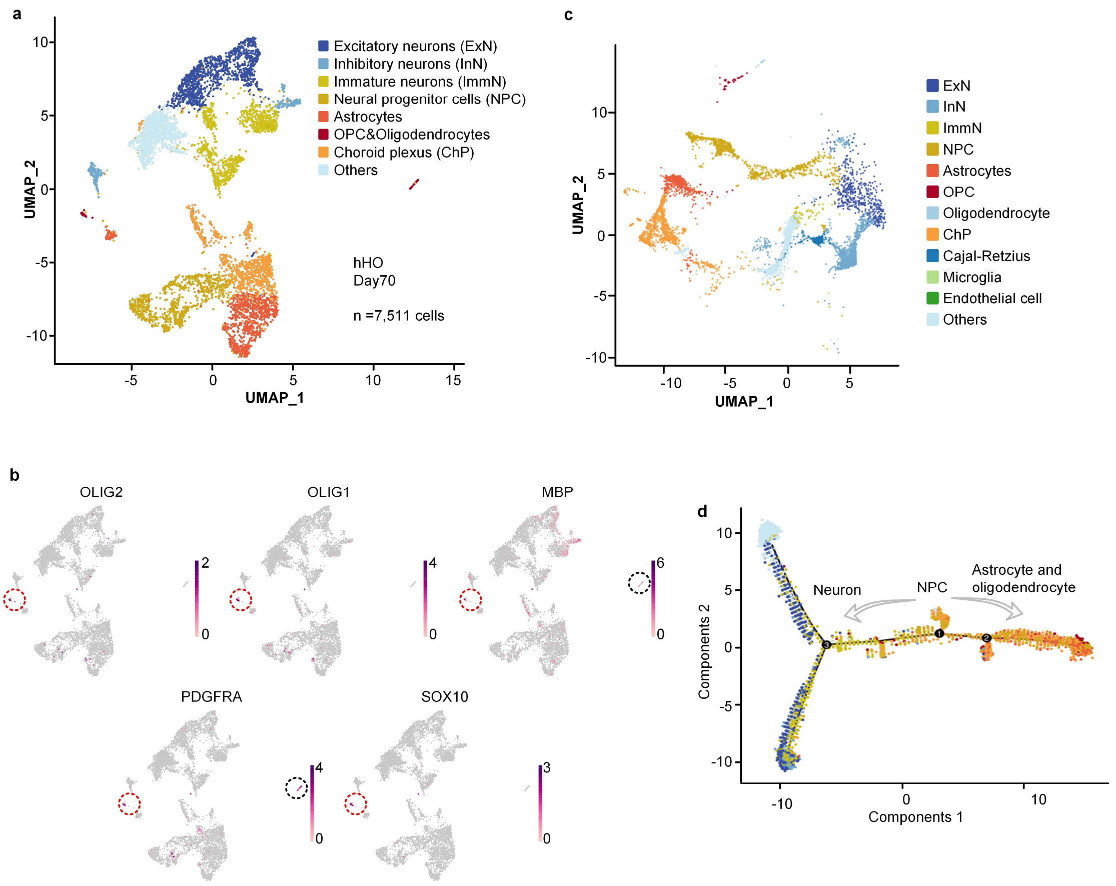

Supplementary Fig. 8 | Transcriptomic signature of day-70 hHOs. a, UMAP visualization of scRNA-seq clusters. Two samples of hHOs had similar cell types, but the dataset at day-81 hHOs was missing the population of cells co-expressing oligodendrocyte markers. **b**, The expression of OPC and oligodendrocyte markers in the dataset of day-70 hHOs. **c**, The distribution of day-70 hHO sample separated from the integrated dataset. **d**, Trajectory tree showing cell lineage relationships of all cells. Arrows show the directions of lineages. The ‘others’ cluster took up one termination in the upper path of neurons.

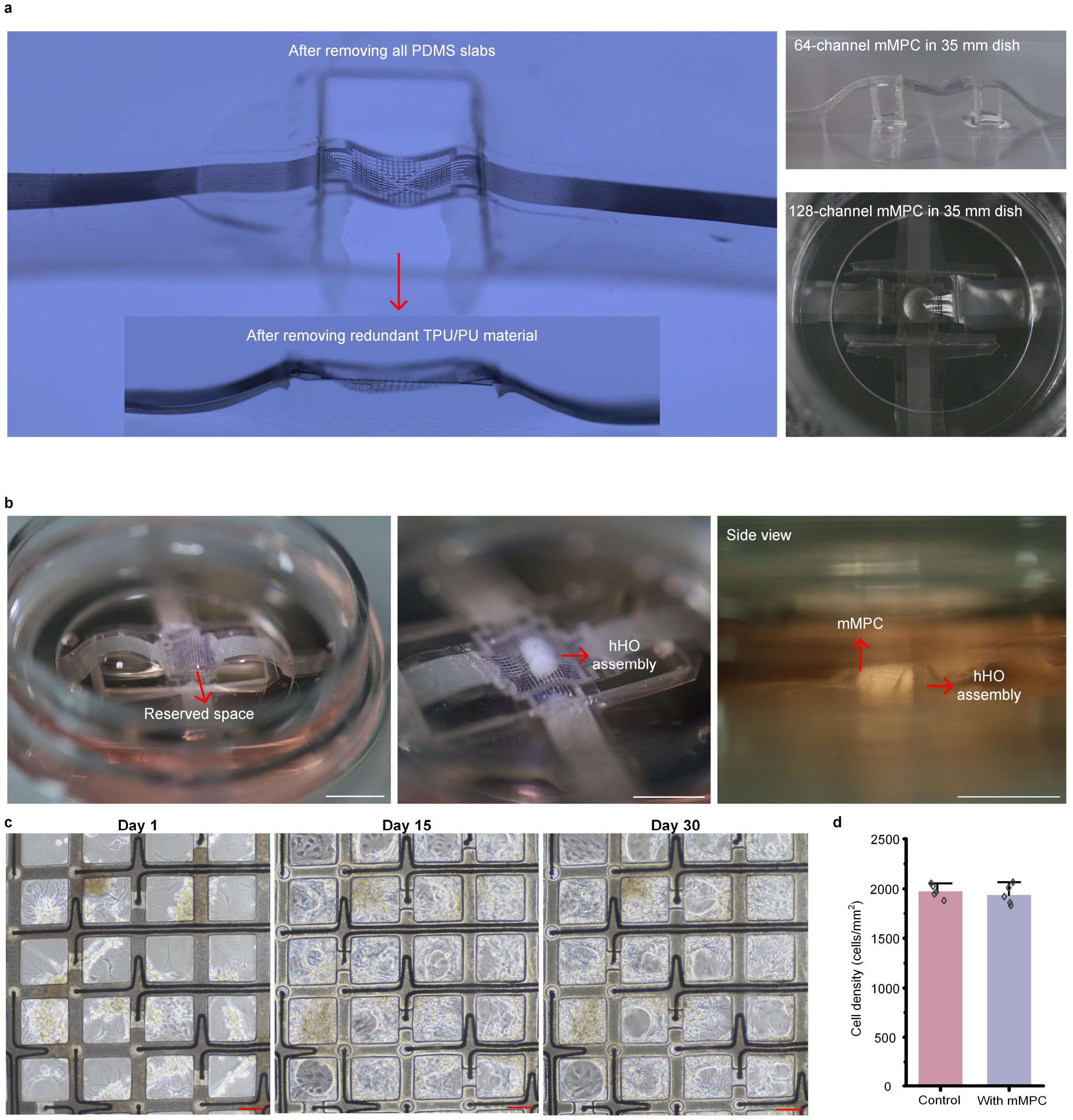

Supplementary Fig. 9 | Assembling of 128-channel mMPC and biocompatibility of mMPC. a, The integrated device coupling the mMPC with culture dishes. (Left): The mesh naturally formed a structure with a slightly concave center after being peeled from the PDMS slabs, which helped to place the hHO in it. The enlarged image also showed that a concave structure was present naturally after cutting off the redundant TPU/PU materials around the mMPC. (Upper right) When assembling the bottom mMPC, we fixed the distance between the two sides so that the bottom mMPC appeared in a "bowl" shape. (Lower Right): Assembly of two-layer mMPCs with the 35-mm dish. The TPU ball in the center was to reserve the space the hHO may need. **b,** (Left) The two-layer mMPCs with culture medium. After removing the TPU ball, some space existed between these two layers. (Middle and right) The top and the side view after placing one hHO assembly into the two-layer mMPC. Scale bar: 5 mm. When the culture solution was just submerged over the hHO assembly, the top mMPC covered the surface of the hHO assembly without significant squeezing. **c,** Biocompatibility. Cells from suckling mice hippocampus grew in the mMPC for 30 days. Scale bar: 100 μm. **d,** Comparison of cell density between cultured neural cells with and without mMPC for 10 days (n = 5, 5 independent measurements using 5 samples).

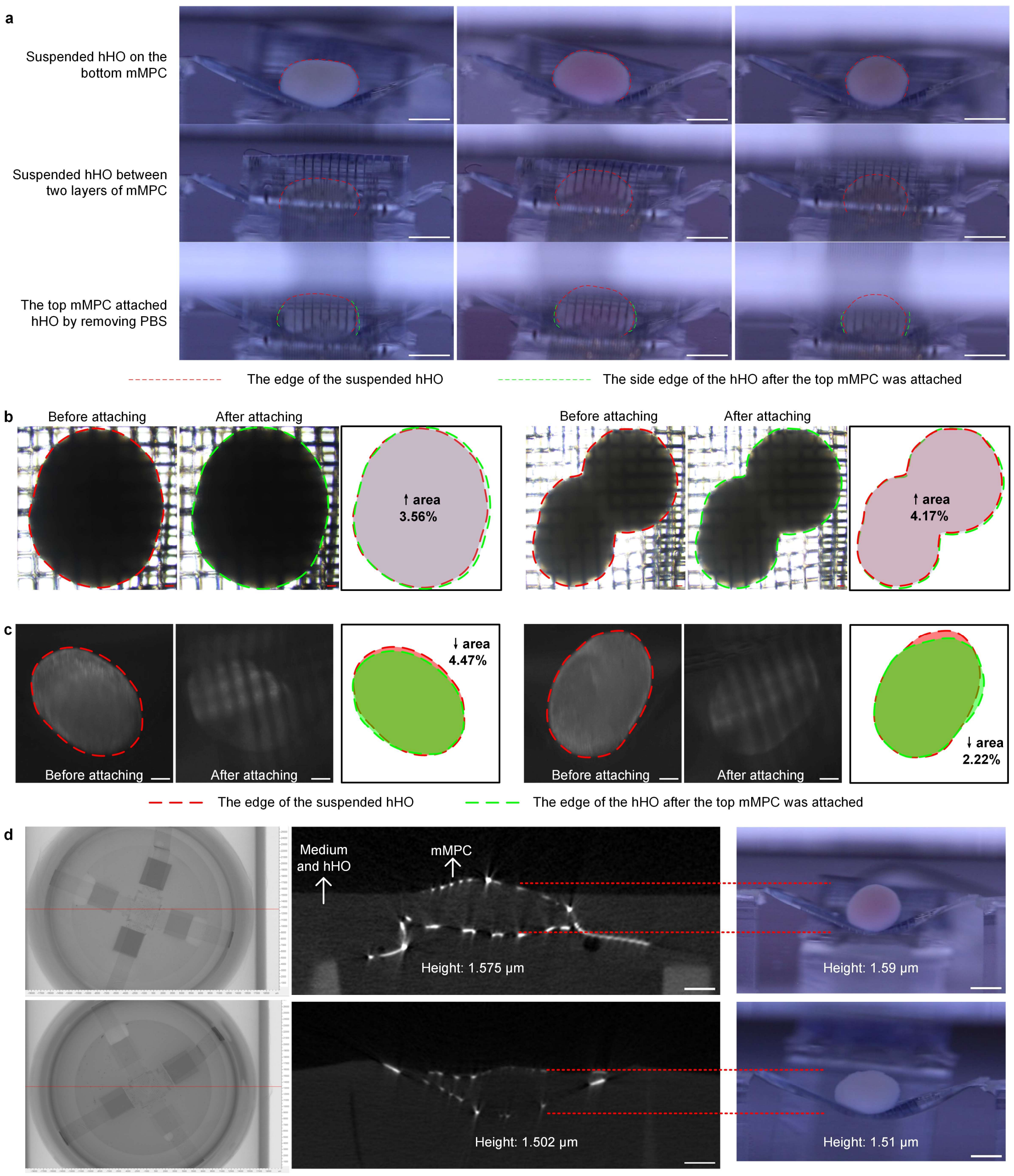

Supplementary Fig. 10 | The deformation of hHOs sandwiched between two mMPCs. a, The side view of hHOs before and after sandwiched between two mMPCs. Scale bar: 1 mm. **b**, The bottom view of hHO and hHO assembly before and after sandwiched between two mMPCs. Scale bar: 100 μm. **c**, The side view of Fluo-8-loaded hHOs before and after sandwiched between two mMPCs. Scale bar: 500 μm. The complete process of Figure **a**-**c** was recorded in Supplementary Movie 4. When the hHO was inserted into the space between the top and bottom mMPCs, the hHO was still free-floating but limited in this space. We could shake it. After slowly removing the medium, the top mMPC was attached to the hHO surface. The hHO was fixed at that location, and we could not shake it. **d**, Micro-CT Images of hHOs sandwiched between two mMPCs. (Left) The hHO tissue could not be distinguished from the medium due to the lack of vascular structures. The mMPC could be captured due to its higher density. (Middle) The cross-section in the red line of the left figure. (Right) The suspended hHO at the bottom mMPC. Scale bar: 1 mm.

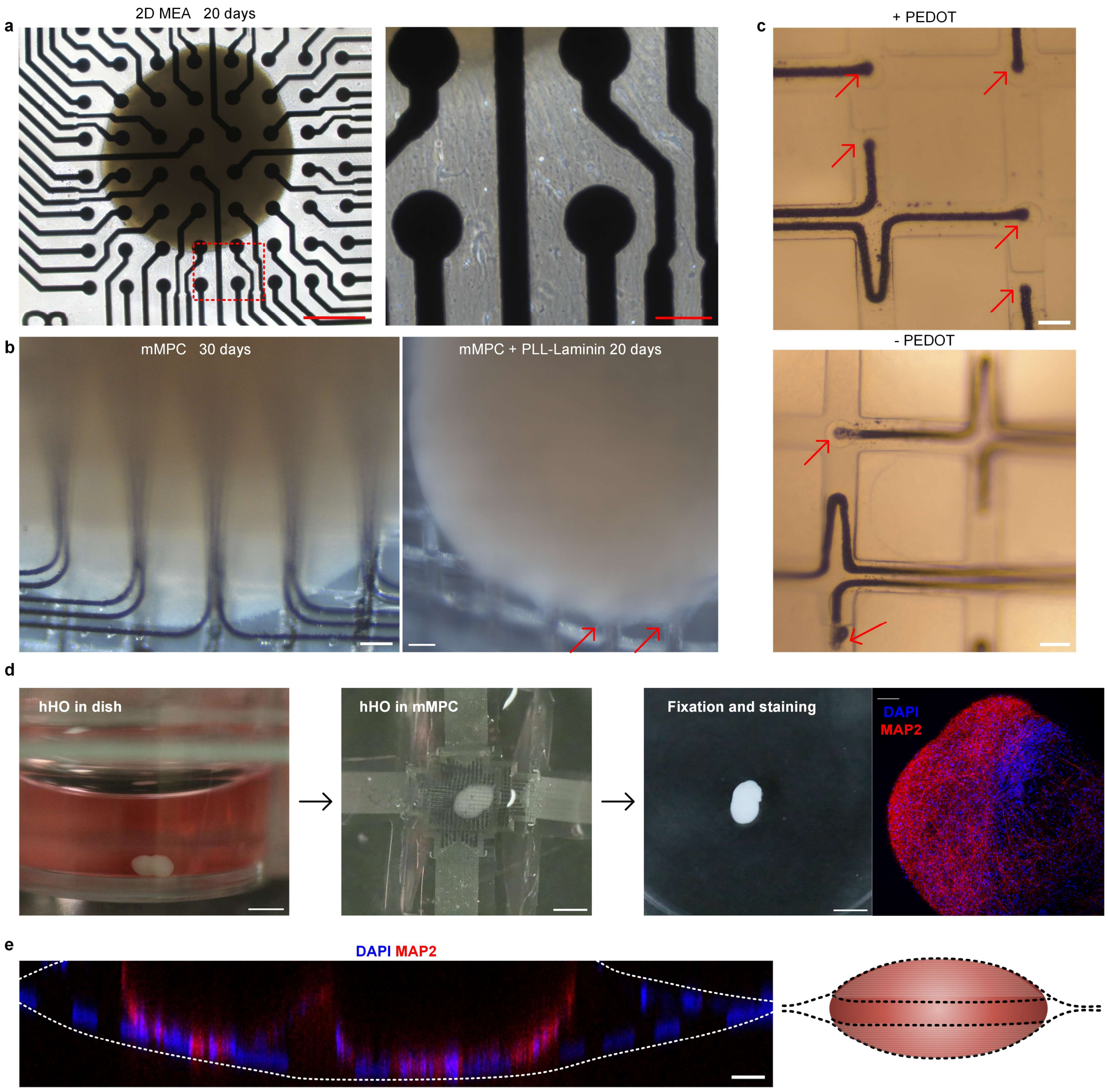

Supplementary Fig. 11 | Long-term change of hHOs in MEA and noninvasive assessment. a, (Left) The hHO on a commercial 2D MEA after culturing for 20 days. Scale bar: 500 μm. (Right) zoom-in view of the red box in the left figure showing the cell migration on the 2D MEA. Scale bar: 100 μm. **b,** (Left) bright-filed images of hHOs on the normal mMPC after cocultured for 30 days and on the PLL-Laminin-coated mMPC for 20 days. Scale bar: 100 μm. **c**, The mMPC electrodes with and without PEDOT coating after culturing the hHO for 2 weeks. The red arrows point to the electrodes. Scale bar: 50 μm. **d**, Non-invasive assessment. The morphology of the same hHO in dish (Left); in the mMPC for 4 days (Middle); after fixation and immunostaining (Right). Scale bar: 100 μm in the staining image and 2 mm in others. **e**, The cross-sectional view showed the attachment boundary between the mMPC and the hHO. Scale bar: 100 μm. There was a certain distance between the two layers.

`

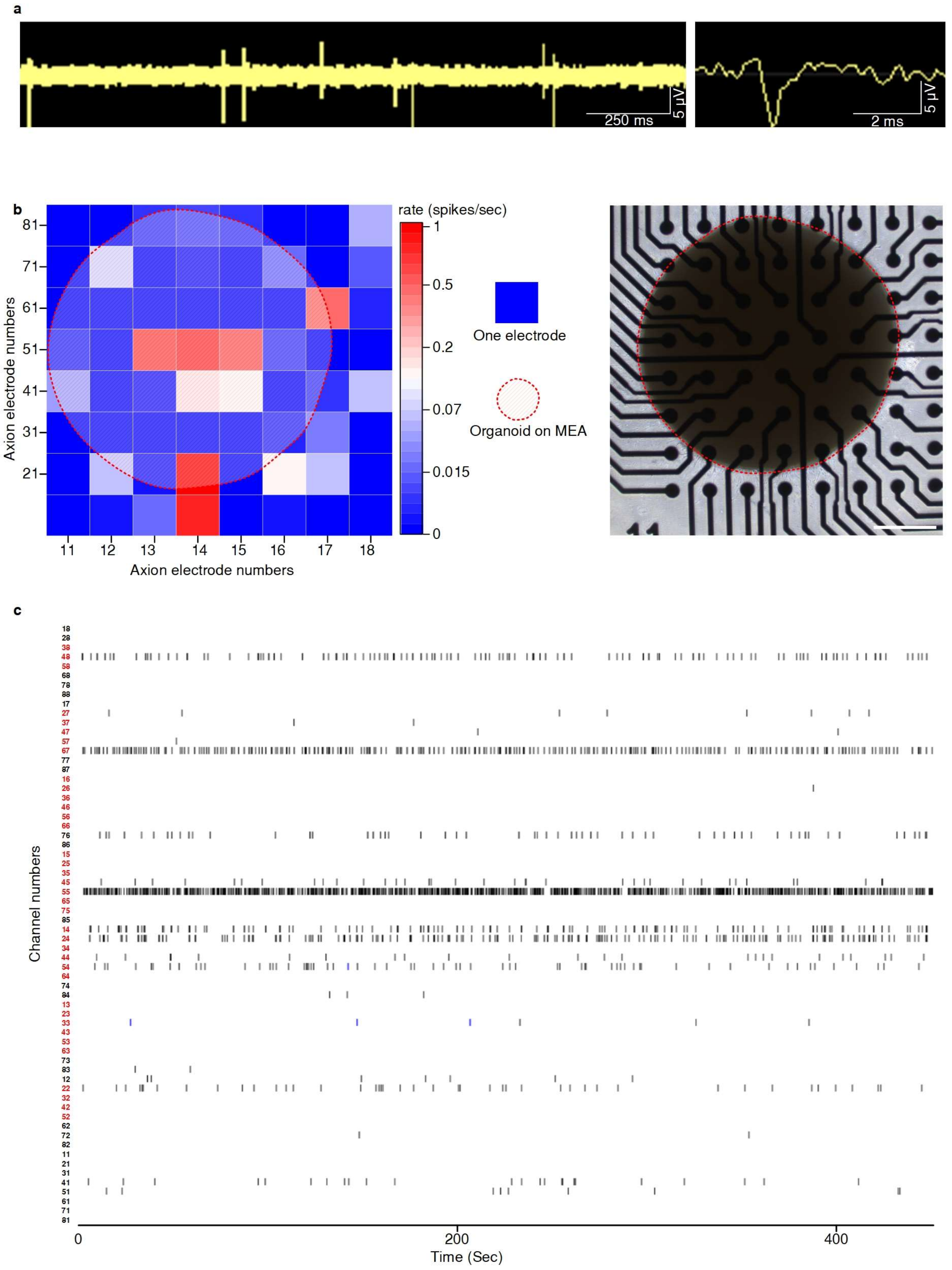

Supplementary Fig. 12 | The neural activities of hHOs placed on 2D MEA. a, The recording of neural signal on the channel of 2D MEA. **b,** (Left) spike rates detected by 64 electrodes. A square represents an electrode and its location. The red dotted circle represented the coverage area of the hHO on the 2D MEA, corresponding to the right figure. (Right) the hHO on the 64-channel MEA. Scale bar: 500 µm. **c,** Raster image of spontaneous spikes occurred in 64 channels of the 2D MEA. The red channel numbers represent those electrodes covered by the hHO. It figured that the more active neural activities were mainly concentrated at these red channel numbers.

Supplementary Table 1 | Active channel numbers in different hippocampal cyb-organoids. Our Plexon system detected and recorded data from up to 32 channels simultaneously. Due to this limitation, detecting four times (128 channels) was necessary, and it was impossible to obtain data from 128 channels simultaneously.

Sample #	CH 1-32	CH 33-64	CH 65-96	CH 97-128	Total active channels
1 assembly	17	18	28	22	85
2 assembly	22	11	13	14	60
3 assembly	12	28	25	9	74
4 individual hHO	18	15	8	3	44
5 individual hHO	22	15	7	10	54

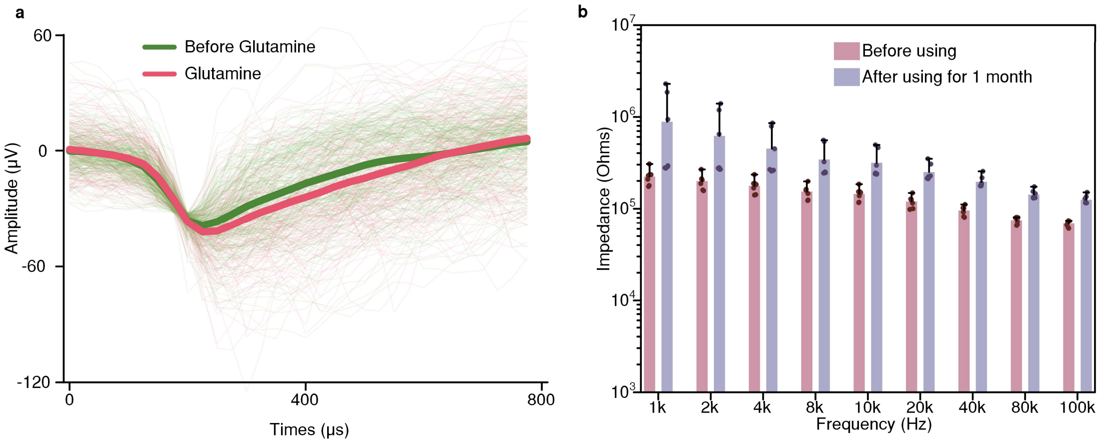

Supplementary Fig. 13 | a, The change of amplitude of glutamine-induced spikes. **b,** The impedance of the mMPC before and after integration with the hHO for 1 month. (n=7, 7 independent electrodes randomly selected from 2 mMPCs; data are presented as median with maxima).

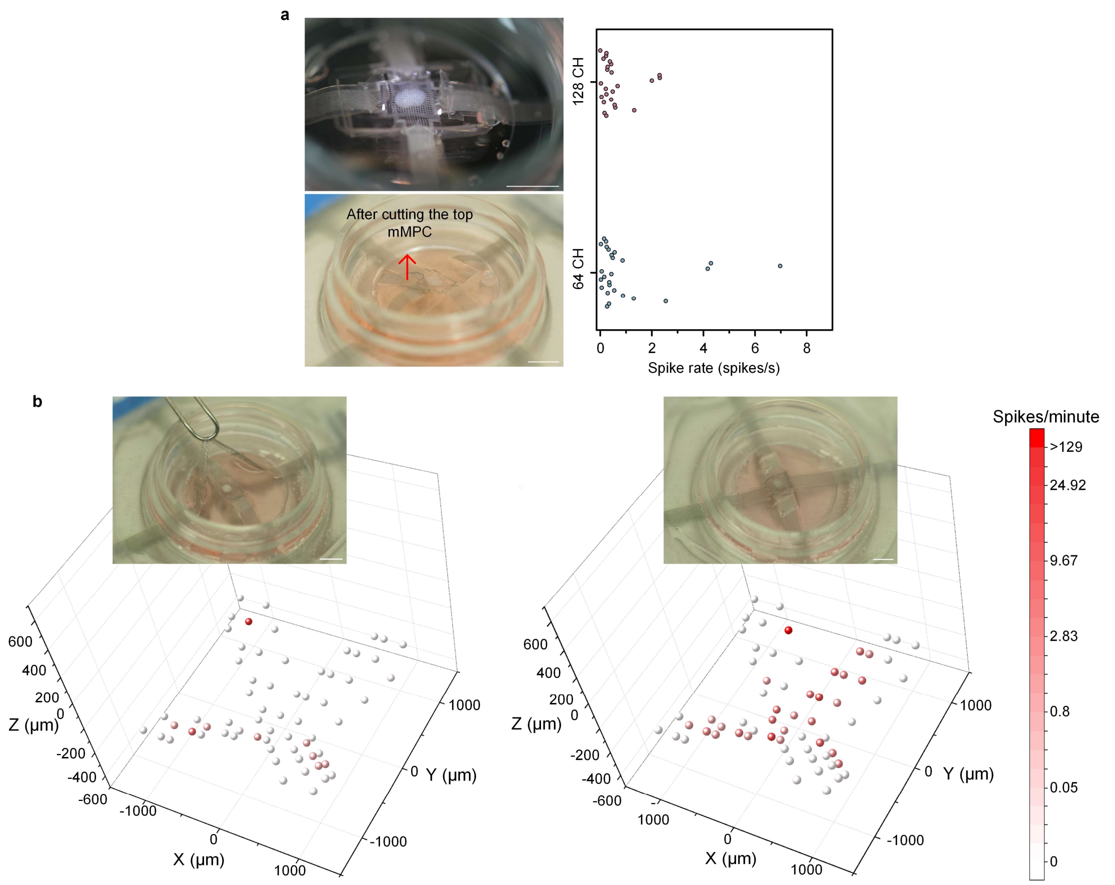

Supplementary Fig. 14 | a. The comparison of spike rates recorded by the bottom mMPC before and after cutting the top mMPC. Scale bar: 5 mm. **b,** The comparison of spike rates recorded by the bottom mMPC before and after attachment of the top mMPC. Scale bar: 5 mm.

Key resources table

Reagents, Chemicals, and Recombinant Proteins

	Source	Catalog Number
mTeSR	STEMCELL Technologies	85850
ReLeSR	STEMCELL Technologies	05872
Accutase	STEMCELL Technologies	07920
DPBS	Corning	21-031-CV
Matrigel	Corning	354277
DMEM/F-12	ThermoFisher	11330032
MEM-NEAA	ThermoFisher	11140050
GlutaMax	ThermoFisher	35050061
Penicillin/Streptomycin	ThermoFisher	10378016
LDN-193189	Sigma	SML0559
SB431542	Abcam	ab120163
Cyclopamine	MCE	HY-17024
XAV939	STEMCELL Technologies	72672
Rock inhibitor	STEMCELL Technologies	72307

KnockOut Serum Replacement	ThermoFisher	10828028
FBS	ThermoFisher	10100147
Neurobasal medium	ThermoFisher	21103049
BMP4	R&D Systems	314-BP-010
CHIR 99021	STEMCELL Technologies	72052
β-Mercaptoethanol	ThermoFisher	21985023
B27 (minus vitamin A)	ThermoFisher	12587010
B27	ThermoFisher	17504044
N2	ThermoFisher	17502-048
BDNF	STEMCELL Technologies	78005.1
GNDF	STEMCELL Technologies	78058.1
Wnt3a	R&D Systems	5036-WN-010/CF
Purmorphamine	STEMCELL Technologies	72202
PBST	ESscience	ES4013
O.C.T	Tissue-Tek	4583
BSA	Sigma	B2064
Sucrose	Macklin	S824459
PFA	Leagene	DF0135
Triton X-100	Sigma	9036-19-5
Neuronal Isolation enzyme (with papain)	ThermoFisher	88285
HBSS	ThermoFisher	88284
Poly-L-lysine solution	Sigma	P4832
Laminin	Sigma	L2020
SU-8 negative photoresist	MicroChem	SU-8 2015
PDMS	Dow	Sylgard 184
GaIn alloy	Beijing Hawk Technology	
98% n-Decyl alcohol	MACKLIN	D806660
TPU	EVERMORE CHEMICAL INDUSTRY	BTE-75A
99.5% DMF	MACKLIN	N807505
Sodium sulfate	MACKLIN	S818055
EDOT	Shanghai yuanye Bio-Technology	S30619
PSS	Sigma	25704-18-1
Fluo-8 AM	Abcam	ab142773

Antibodies

Antibody	Company	Catalog numbers	Dilution	Source
Anti-LEF1	Abcam	ab137872	1:500	Rabbit monoclonal
Anti-SOX2	Abcam	ab92494	1:100	Rabbit monoclonal
Anti-PAX6	Proteintech	12323-1-AP	1:100	Rabbit polyclonal
Anti-PAX6	Abcam	ab78545	1:100	Mouse monoclonal
Anti-ZBTB20	Proteintech	23987-1-AP	1:100	Rabbit polyclonal
Anti-Olig2	Abcam	ab109186	1:100	Rabbit monoclonal
Anti-FOXG1	Abcam	ab196868	1:100	Rabbit monoclonal

Anti-FOXG1	Genetex	GTX134018	1:1000	Rabbit polyclonal
Anti-HOPX	Proteintech	11419-1-AP	1:300	Rabbit polyclonal
Anti-HOPX	Thermo Fisher	PA590538	1:200	Rabbit polyclonal
Anti-Prox1	R&D Systems	AF2727	1:100	Goat polyclonal
Anti-Prox1	Abcam	ab199359	1:500	Rabbit monoclonal
Anti-MAP2	Invitrogen	MA512826	1:1000	Mouse monoclonal
Anti-Nestin	Abcam	ab6320	1:1000	Mouse monoclonal
Anti-NeuN	Genetex	GTX132974-S	1:500	Rabbit polyclonal
Anti-GFAP	Genetex	GTX108711	1:1000	Rabbit polyclonal
Anti-gammaTubulin	Abcam	ab27074	1:1000	Mouse monoclonal
Anti-TAU	Abcam	ab92676	1:500	Rabbit monoclonal
Anti-TTR	Proteintech	11891-1-AP	1:100	Rabbit polyclonal
Anti-SULF2	Abcam	ab232835	1:200	Rabbit polyclonal
Anti-SEMA5A	EpigenTek	A64642-020	1:500	Rabbit polyclonal
Goat Anti-Rabbit IgG H&L (Alexa Fluor 488)	Abcam	ab150077	1:1000	
Goat Anti-Rabbit IgG H&L (Alexa Fluor 647)	Abcam	ab150083	1:1000	
Goat-Mouse 488	Genetex	GTX213111-04	1:1000	
Goat Anti-Mouse IgG H&L (Alexa Fluor 647)	Abcam	ab150115	1:1000	
Goat Anti-Rabbit IgG H&L (Alexa Fluor 594)	Abcam	ab150080	1:1000	
Phalloidin-iFluor 555	Abcam	ab176756	1:1000	

Critical Commercial Assays

Pierce Primary Neuron Isolation Kit	Thermo Fisher	88280

Cell lines

IMR90-4	WiCell	WB65316
Foreskin-4	Wicell	WB66699
hiPSC	Cellapy	hiPSC-U1

Software

ImageJ	NIH
Imaris viewer	Bitplane
Origin	OriginLab
Visio	Microsoft
AxIS Navigator	Axion
NeuralMetric Tool	Axion
NIS viewer	Nikon
Corel VideoStudio	Corel
Loupe Browser	10x Genomics

AutoCAD	Autodesk
OmniPlex	Plexon
Offline Sorter	Plexon
NeuroExplorer.	Plexon
CT-Analyser	Bruker
CTvox	Bruker

Primers for qPCR

GAPDH-forward	5‘-TCAAGAAGGTGGTGAAGCAG-3’
GAPDH-reverse	5‘-CGCTGTTGAAGTCAGAGGAG-3’
ZBTB20-forward	5‘-TGGCACCCAGATCGAGAAC-3’
ZBTB20-reverse	5‘-GTGGAACCGCATTTTTCCCC-3’
TTR-forward	5‘-ATCCAAGTGTCCTCTGATGGT-3’
TTR-reverse	5‘-GCCAAGTGCCTTCCAGTAAGA-3’
PROX1-forward	5‘-GACTTTGAGGTTCCAGAGAGA-3’
PROX1-reverse	5‘-TGTAGGCAGTTCGGGGATTTG-3’
Wnt3a-forward	5’-GATGGTGGTGGAGAAGCAC-3’
Wnt3a-reverse	5’-GTGGGCACCTTGAAGTAGGT-3’
