# Supplementary material for: Three-dimensional liquid metal-based neuro-interfaces for human hippocampal organoids
Source: Nat Commun. 2024 May 14;15:4047. doi: 10.1038/s41467-024-48452-5 (PMC11094048; doi:10.1038/s41467-024-48452-5)
Supplement: Supplementary file 3 — Description of Additional Supplementary Files [file 41467_2024_48452_MOESM3_ESM.pdf]

Description of Additional Supplementary Files

File Name: Supplementary Movie 1
Description: Stretchability of mMPC.

File Name: Supplementary Movie 2
Description: Twist of mMPC.

File Name: Supplementary Movie 3
Description: Using process.

File Name: Supplementary Movie 4
Description: The bottom and side views of hHOs after sandwiching between mMPCs.

File Name: Supplementary Movie 5
Description: The hHO sandwiched between mMPCs captured by micro-CT

File Name: Supplementary Movie 6
Description: Spike rates in 128 channels.
