# Supplementary material for: Three-dimensional liquid metal-based neuro-interfaces for human hippocampal organoids
Source: Nat Commun. 2024 May 14;15:4047. doi: 10.1038/s41467-024-48452-5 (PMC11094048; doi:10.1038/s41467-024-48452-5)
Supplement: Supplementary file 10 — Reporting Summary [file 41467_2024_48452_MOESM10_ESM.pdf]

nature portfolio

Corresponding author(s): Xingyu Jiang, NCOMMS-23-19904B-Z

Last updated by author(s): Apr 23, 2024

Reporting Summary

Nature Portfolio wishes to improve the reproducibility of the work that we publish. This form provides structure for consistency and transparency in reporting. For further information on Nature Portfolio policies, see our Editorial Policies and the Editorial Policy Checklist.

Statistics

For all statistical analyses, confirm that the following items are present in the figure legend, table legend, main text, or Methods section.

n/a	Confirmed	
☐	☒	The exact sample size (*n*) for each experimental group/condition, given as a discrete number and unit of measurement
☐	☒	A statement on whether measurements were taken from distinct samples or whether the same sample was measured repeatedly
☐	☒	The statistical test(s) used AND whether they are one- or two-sided<br>*Only common tests should be described solely by name; describe more complex techniques in the Methods section.*
☒	☐	A description of all covariates tested
☒	☐	A description of any assumptions or corrections, such as tests of normality and adjustment for multiple comparisons
☐	☒	A full description of the statistical parameters including central tendency (e.g. means) or other basic estimates (e.g. regression coefficient) AND variation (e.g. standard deviation) or associated estimates of uncertainty (e.g. confidence intervals)
☐	☒	For null hypothesis testing, the test statistic (e.g. *F*, *t*, *r*) with confidence intervals, effect sizes, degrees of freedom and *P* value noted<br>*Give P values as exact values whenever suitable.*
☒	☐	For Bayesian analysis, information on the choice of priors and Markov chain Monte Carlo settings
☒	☐	For hierarchical and complex designs, identification of the appropriate level for tests and full reporting of outcomes
☒	☐	Estimates of effect sizes (e.g. Cohen's *d*, Pearson's *r*), indicating how they were calculated

Our web collection on statistics for biologists contains articles on many of the points above.

Software and code

Policy information about availability of computer code

Data collection	NIS-Elements (Nikon), Image-Pro (Hitachi), AxIS Navigator (Axion), OmniPlex (Plexon), Nova (Metrohm Autolab),10X Genomics, Skyscan 1276 (Bruker)
Data analysis	Imaris viewer (Bitplane), NeuralMetric Tool (Axion), Offline Sorter and NeuroExplorer (Plexon), Loupe (10X Genomics), Origin, ImageJ, CT-Analyser, CTvox

For manuscripts utilizing custom algorithms or software that are central to the research but not yet described in published literature, software must be made available to editors and reviewers. We strongly encourage code deposition in a community repository (e.g. GitHub). See the Nature Portfolio guidelines for submitting code & software for further information.

Data

Policy information about availability of data

All manuscripts must include a data availability statement. This statement should provide the following information, where applicable:

- Accession codes, unique identifiers, or web links for publicly available datasets
- A description of any restrictions on data availability
- For clinical datasets or third party data, please ensure that the statement adheres to our policy

The processed 10X Genomics datasets generated in this study have been deposited in the Gene Expression Omnibus (GEO) database under accession code GSE264363. All other data generated in this study are provided in the Supplementary information files or in the Source Data file, or from the corresponding author upon request. Source data are provided with this paper.

Research involving human participants, their data, or biological material

Policy information about studies with human participants or human data. See also policy information about sex, gender (identity/presentation), and sexual orientation and race, ethnicity and racism.

Reporting on sex and gender	This study did not involve human participants, so this information has not been collected.
Reporting on race, ethnicity, or other socially relevant groupings	This study did not involve human participants, so this information has not been collected.
Population characteristics	This study did not involve human participants.
Recruitment	This study did not involve human participants.
Ethics oversight	This study did not involve human participants.

Note that full information on the approval of the study protocol must also be provided in the manuscript.

Field-specific reporting

Please select the one below that is the best fit for your research. If you are not sure, read the appropriate sections before making your selection.

- [x] Life sciences
- [ ] Behavioural & social sciences
- [ ] Ecological, evolutionary & environmental sciences

For a reference copy of the document with all sections, see nature.com/documents/nr-reporting-summary-flat.pdf

Life sciences study design

All studies must disclose on these points even when the disclosure is negative.

Sample size	Sample sizes were chosen on the basis of previous experience and standards in the field.
Data exclusions	Data were not excluded from analysis.
Replication	All experiments were replicated multiple times, and all results were replicable. Organoids were derived from different stem cell lines and generated in many batches. Data from single-cell RNA sequencing were collected from 4 organoids. Neural signal recordings were repeated in different organoid-mesh MPC complexes. The characterization of electronics was repeated many times and measured in different devices. The deformation of organoids sandwiched between two-layer mesh MPCs was measured with many methods, using multiple organoids.
Randomization	All devices were tested randomly, and organoids were selected randomly.
Blinding	Blinding was not possible as experimental conditions were evident from the image data. Quantifications were performed using computational pipeline applied equally to all conditions, including experimental groups and control groups.

Reporting for specific materials, systems and methods

We require information from authors about some types of materials, experimental systems and methods used in many studies. Here, indicate whether each material, system or method listed is relevant to your study. If you are not sure if a list item applies to your research, read the appropriate section before selecting a response.

Materials & experimental systems

n/a	Involved in the study	
	x	Antibodies
	x	Eukaryotic cell lines
x		Palaeontology and archaeology
x		Animals and other organisms
x		Clinical data
x		Dual use research of concern
x		Plants

Methods

n/a	Involved in the study	
x		ChIP-seq
x		Flow cytometry
x		MRI-based neuroimaging

Antibodies

Antibodies used

Antibodies used in this study and their information were listed in Supplementary information. Include:
Anti-LEF1, Abcam, ab137872, 1:500, Rabbit monoclonal;
Anti-SOX2, Abcam, ab92494,1:100, Rabbit monoclonal;
Anti-PAX6, Proteintech, 12323-1-AP, 1:100, Rabbit polyclonal;
Anti-PAX6, Abcam, ab78545, 1:100, Mouse monoclonal;
Anti-ZBTB20, Proteintech, 23987-1-AP, 1:100, Rabbit polyclonal;
Anti-Olig2, Abcam, ab109186, 1:100, Rabbit monoclonal;
Anti-FOXG1, Abcam, ab196868, 1:100, Rabbit monoclonal;
Anti-FOXG1, Genetex, GTX134018, 1:1000, Rabbit polyclonal;
Anti-HOPX, Proteintech, 11419-1-AP, 1:300, Rabbit polyclonal;
Anti-HOPX, Thermo Fisher , PA590538, 1:200, Rabbit polyclonal;
Anti-Prox1, R&D Systems, AF2727, 1:100, Goat polyclonal;
Anti-Prox1, Abcam, ab199359, 1:500, Rabbit monoclonal;
Anti-MAP2, Invitrogen, MA512826, 1:1000, Mouse monoclonal;
Anti-Nestin, Abcam, ab6320, 1:1000, Mouse monoclonal;
Anti-NeuN, Genetex, GTX132974-S, 1:500, Rabbit polyclonal;
Anti-GFAP, Genetex, GTX108711, 1:1000, Rabbit polyclonal;
Anti-gammaTubulin, Abcam, ab27074, 1:1000, Mouse monoclonal;
Anti-TAU Abcam, ab92676, 1:500, Rabbit monoclonal;
Anti-TTR, Proteintech, 11891-1-AP, 1:100, Rabbit polyclonal;
Anti-SULF2, Abcam, ab232835, 1:200, Rabbit polyclonal;
Anti-SEMA5A, EpigenTek, A64642-020, 1:500, Rabbit polyclonal;
Goat Anti-Rabbit IgG H&L (Alexa Fluor 488), Abcam, ab150077 , 1:1000;
Goat Anti-Rabbit IgG H&L (Alexa Fluor 647), Abcam, ab150083 , 1:1000;
Goat-Mouse 488, Genetex, GTX213111-04, 1:1000;
Goat Anti-Mouse IgG H&L (Alexa Fluor 647), Abcam, ab150115, 1:1000;
Goat Anti-Rabbit IgG H&L (Alexa Fluor 594), Abcam, ab150080 , 1:1000;
Phalloidin-iFluor 555, Abcam, ab176756, 1:1000.

Validation

All antibodies have been validated by Abcam, Proteintech, Genetex, R&D System, Thermo Fisher, and EpigenTek by demonstrating immunofluorescence on cells, as stated on their product pages (see website).

Eukaryotic cell lines

Policy information about cell lines and Sex and Gender in Research

Cell line source(s)

iPS (Foreskin)-4 (Human, WiCell WB66699, Donor male);
iPS (IMR90)-4 (Human, WiCell WB65316, Donor female);
hiPSC (Human, CellApy, Donor male)

Authentication

Two cell lines from WiCell were authenticated via Chromosome analysis. The testing was performed by WiCell.
HiPSC from CellApy was authenticated via Chromosome analysis and immunofluorescence (OCT4\SOX4\NANOG\SSEA4 \TRA-1-60\TRA-1-81). The testing was performed by CellApy.

Mycoplasma contamination

All cell lines tested negative for mycoplasma contamination via mycoplasma detection assay. The testing was performed by WiCell and CellApy, respectively.

Commonly misidentified lines
(See ICLAC register)

No commonly misidentified cell lines were used.

Plants

Seed stocks	This study did not involve plants.
Novel plant genotypes	This study did not involve plants.
Authentication	This study did not involve plants.
